# Crosstalk in competing endogenous RNA network reveals the complex molecular mechanism underlying lung cancer

**DOI:** 10.18632/oncotarget.20441

**Published:** 2017-08-24

**Authors:** Xiang Jin, Yinghui Guan, Hui Sheng, Yang Liu

**Affiliations:** ^1^ Department of Respiration, The First Hospital of Jilin University, Changchun City, Jilin Province, 130021, China

**Keywords:** crosstalk, ceRNA network, lung cancer, molecular mechanism, circRNA-seq

## Abstract

We investigated the transcriptional mechanism underlying lung cancer development. RNA sequencing analysis was performed on blood samples from lung cancer cases and healthy controls. Differentially expressed microRNAs (miRNAs), circular RNAs (circRNAs), mRNAs (genes), and long non-coding RNAs (lncRNA) were identified, followed by pathway enrichment analysis. Based on miRNA target interactions, a competing endogenous network was established and significant nodes were screened. Differentially expressed transcriptional factors were retrieved from the TRRUST database and the transcriptional factor regulatory network was constructed. The expression of 59 miRNAs, 18,306 genes,232 lncRNAs, and 292 circRNAs were greatly altered in patients with lung cancer. miRNAs were closely associated with cancer-related pathways, such as pathways in cancer, colorectal cancer, and transcriptional misregulation in cancer. Two novel pathways, olfactory transduction and neuroactive ligand-receptor interactions, were significantly enriched by differentially expressed genes. The competing endogenous RNA network revealed 5 hub miRNAs. Hsa-miR-582-3p and hsa-miR-582-5p were greatly enriched in the Wnt signaling pathway. Hsa-miR-665 was closely related with the MAPK signaling pathway. Hsa-miR-582-3p and hsa-miR-582-5p were also present in the TF regulatory network. Transcriptional factors of WT1 (wilms tumor 1) and ETV1 (ETS variant 1) were regulated by hsa-miR-657 and hsa-miR-582-5p, respectively, and controlled androgen receptor gene expression. miR-582-5p, miRNA-582-3p, and miR-657 may play critical regulatory roles in lung tumor development. Our work may explore new mechanism of lung cancer and aid the development of novel therapy.

## INTRODUCTION

Lung cancer has a high incidence and mortality and threatens public health worldwide. Recent data have suggested that lung cancer is the leading cause of cancer-related deaths [1]. Approximately 1.82 million people are estimated to be affected by lung cancer, resulting in 1.67 million cancer deaths in 2012 worldwide [1]. Lung cancer is caused by the combined effects of genetic and environmental factors. Despite progress in understanding the pathogenesis of lung cancer, the prognosis of lung cancer remains poor with a 5-year survival of less than 15% [2]. With the development of molecular biology techniques, novel therapeutic targets and prognostic biomarkers for lung carcinogenesis have been discovered.

MicroRNAs (miRNAs) are a class of small non-protein-coding RNAs that play regulatory roles in gene expression by binding to messenger RNAs (mRNAs) [3] to mediate various biological functions [4]. miRNA binding to mRNA comprises the minimal competing endogenous RNA (ceRNA) network. In addition to miRNAs, transcription factors (TFs) regulate gene expression to control protein output in the ceRNA network [5]. ceRNAs, as coding or non-coding RNAs, share the recognition elements of miRNAs and increase the complexity of the miRNA-based regulation network [6]. miRNAs play critical roles in disease or cancer development by participating in multiple networks [7].

Circular RNAs (circRNAs) are a class of non-coding RNAs and are abundant in the cytoplasm; they are characterized by acting as miRNA sponges or ceRNAs [8]. CircRNAs can regulate gene expression in mammals [8] and are differentially expressed based on cell type and developmental stage [9]. circRNAs have shown potential in the diagnosis and targeted therapy of cancers [10]. However, the role of circRNAs in lung cancer has not been widely examined. CircRNAs are abundant in exosomes, and the sorting of circRNAs to exosomes is related to changes in miRNAs, which prompted us to explore the relationship between circRNAs and miRNAs in lung cancer.

In this study, we applied RNA sequencing (RNA-seq) to explore the transcriptional landscape of the peripheral blood in patients with lung cancer and healthy controls. Differentially expressed (DE) miRNAs and DE circRNAs were filtered in lung cancer cases and a ceRNA network was constructed by combining information for miRNA, mRNA, circRNA, and lncRNAs. Our results provide novel insight into the complex molecular mechanisms of the miRNA-mediated gene regulatory network.

## RESULTS

### Profiling of miRNAs and circRNAs in blood samples of lung cancer and healthy controls

We characterized the miRNA and circRNA transcripts by RNA-seq from 3 pooled control samples and 3 cancer samples, respectively. Each sample was sequenced and approximately 505 million raw reads were obtained by circRNA-seq and 158 million raw data reads by miRNA-seq. After the clean reads were mapped to the human reference genome, we obtained 1028 miRNAs and 43,599 circRNAs (containing 12,857 lncRNAs).

**Figure 1:**
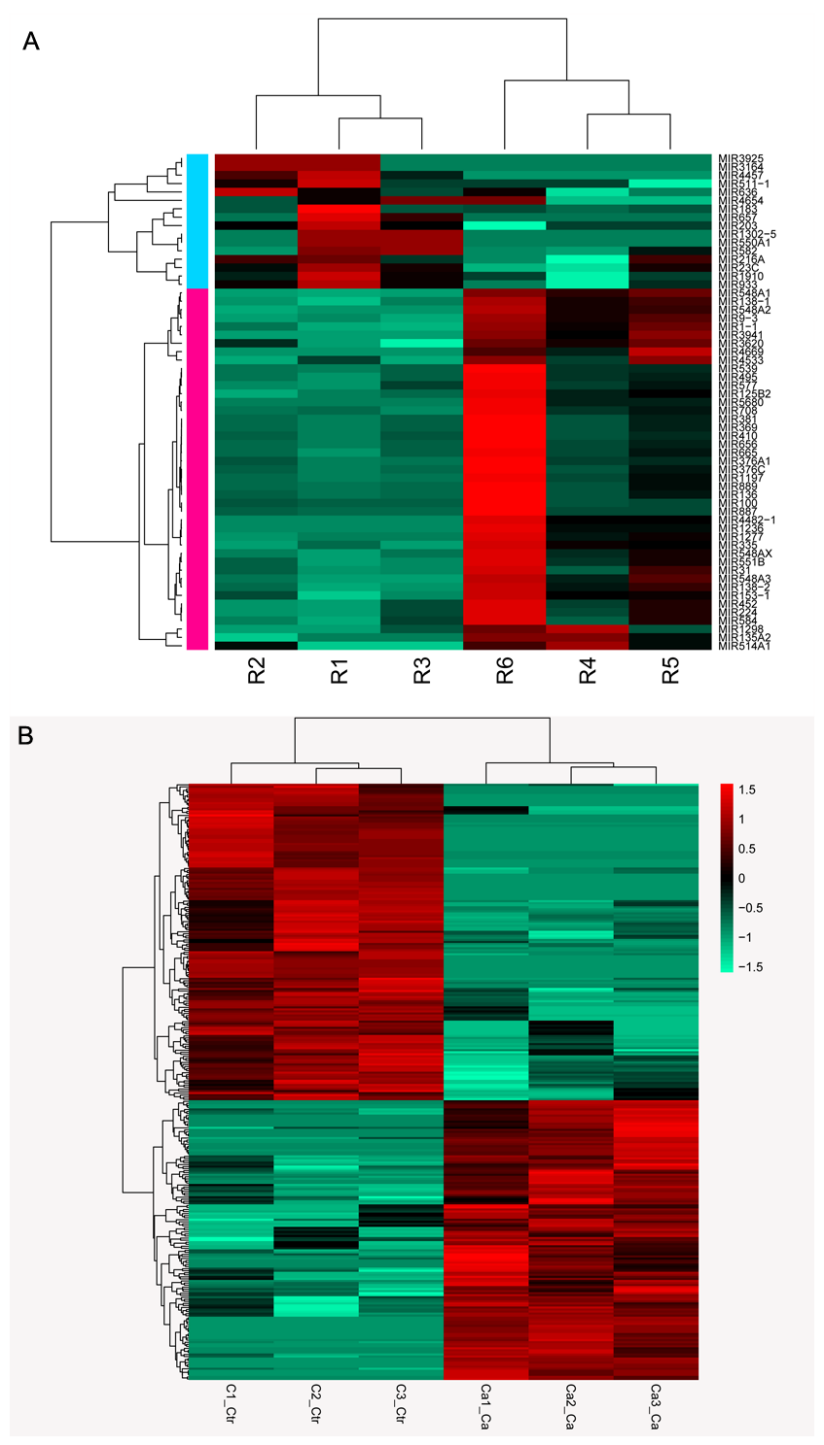
Heatmap of RNA sequencing data **(A)** Expression profiles of differentially expressed miRNAs in patients and controls; **(B)** Expression profiles of differentially expressed circRNAs in patients and controls

DE-miRNAs were analyzed using the limma package, which yielded 59 miRNAs of interest, including 16 up-regulated and 43 down-regulated miRNAs compared to those in healthy controls. Based on circRNA-seq, we obtained 292 DE circRNAs, 232 DE lncRNAs, and 18,306 DE genes. The relative expression levels of DE miRNAs and DE circRNAs in lung cancer samples are depicted in Figure 1.

### Significant pathways enriched by DE genes and DE miRNAs

**Figure 2:**
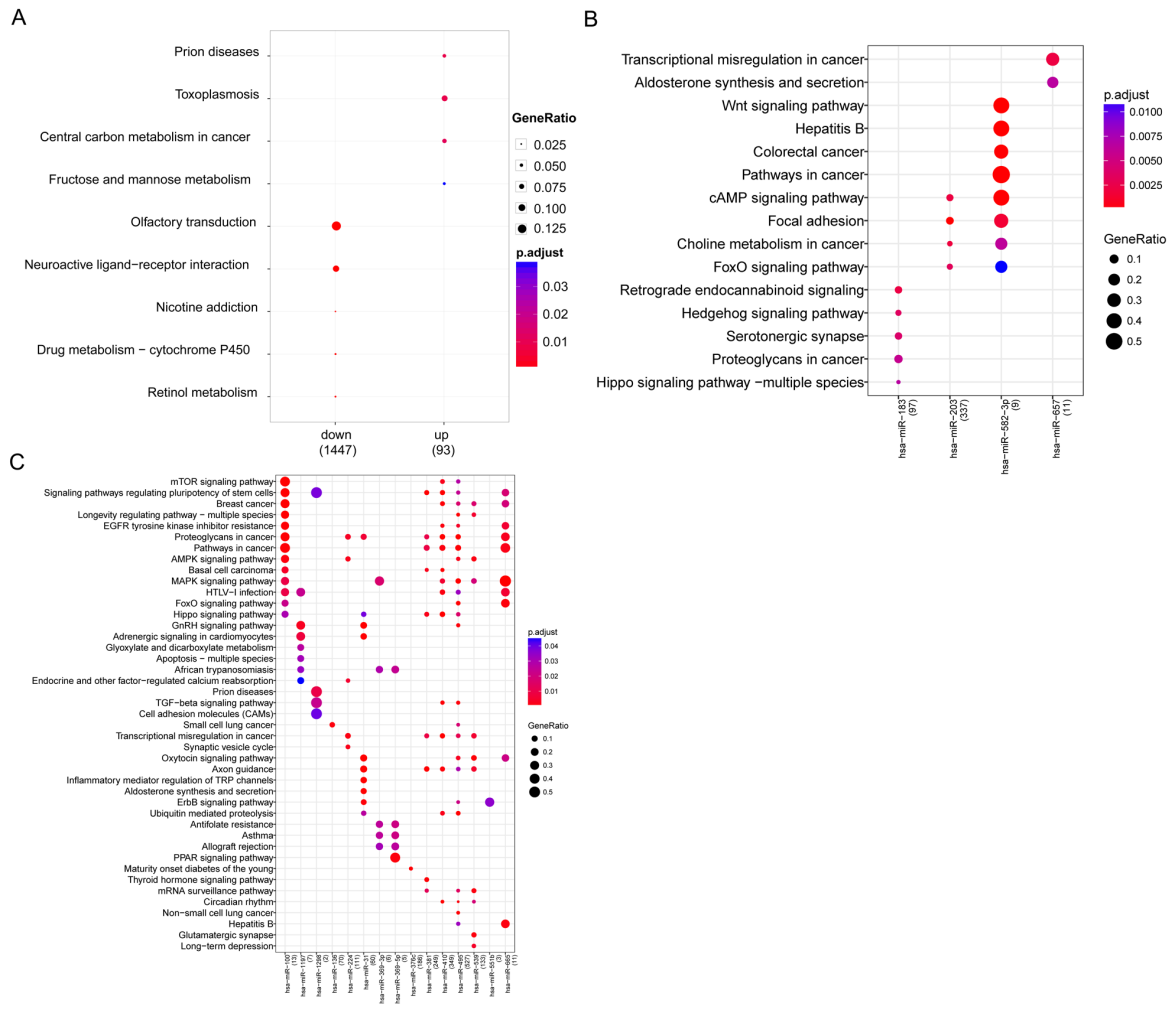
Bubble diagram of significant enrichment of pathways **(A)** Pathways enriched by up- and down-regulated genes; **(B)** Significant pathways for up-regulated miRNAs; **(C)** Significant pathways for down-regulated miRNAs.

Pathway enrichment analysis reveals the genetic determinants and dysregulated pathways underlying lung cancer progression. Pathway enrichment analysis was conducted for up- and down-regulated DE genes. Up-regulated genes were significantly enriched in four pathways: prion diseases, toxoplasmosis, central carbon metabolism in cancer, and fructose and mannose metabolism. Pathways of neuroactive ligand-receptor interaction, nicotine addiction, and drug metabolism-cytochrome P450 were perturbed by down-regulated genes (Figure 2A).

DE miRNA analysis revealed significant enrichment in 4 up-regulated miRNAs and 15 down-regulated miRNAs. Up-regulated miR-203 was closely related to cancer-related pathways such as colorectal cancer. Additionally, miR-203 was related to the cAMP signaling pathway, focal adhesion, and FoxO signaling pathway, as well as miR-203. In addition, miR-657 was closely associated with transcriptional misregulation in cancer, aldosterone synthesis, and secretion, while miR-183 was predicted to be related to retrograde endocannabinoid signaling and the hedgehog signaling pathway (Figure 2B). Conserved targets of down-regulated miRNAs were greatly enriched in the MAPK signaling pathway, cancer-related pathways, proteoglycans in cancer, and signaling pathways regulating the pluripotency of stem cells (Figure 2C).

### CeRNA network

**Figure 3:**
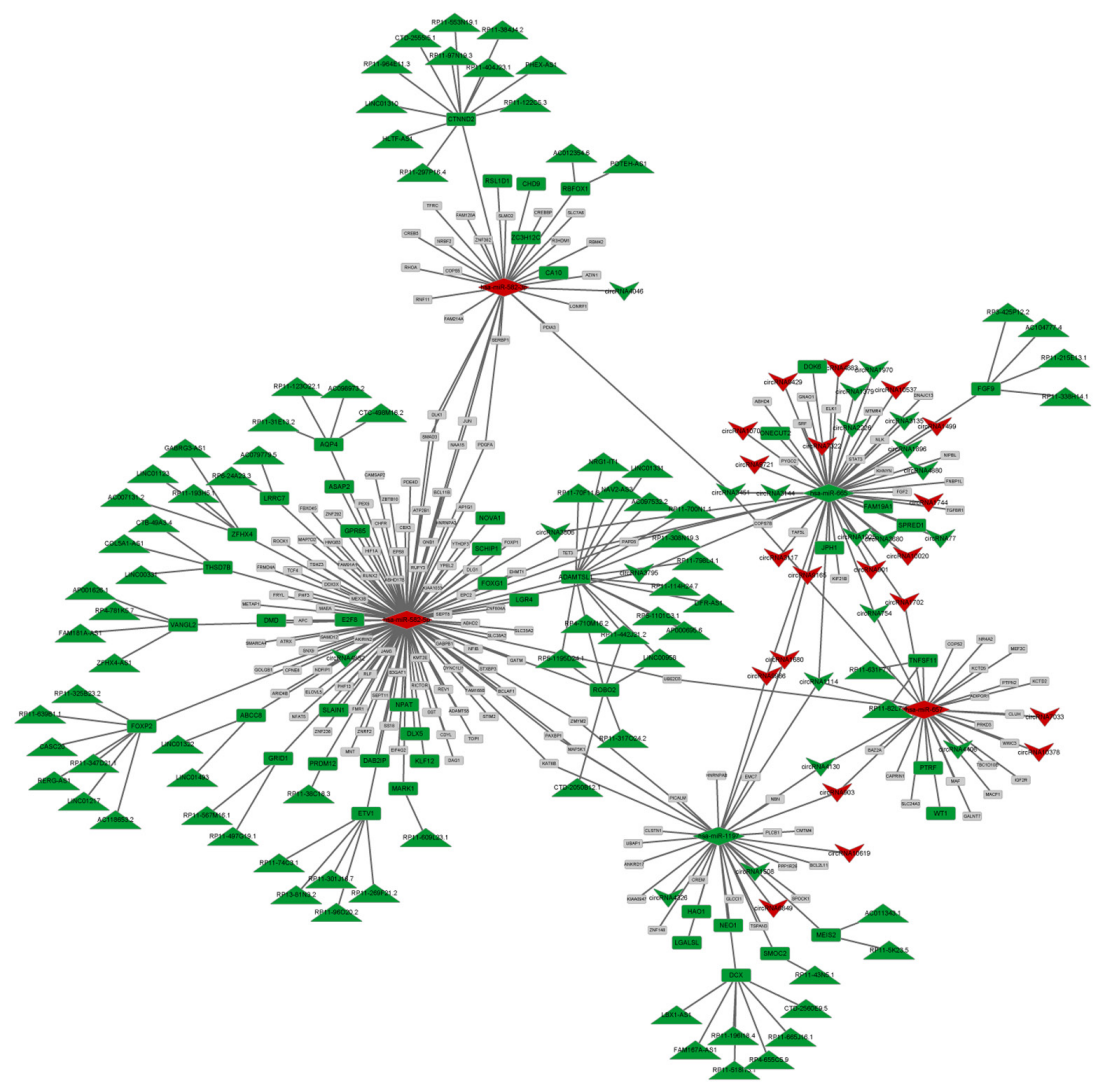
CeRNA network combined with miRNA, circRNA, lncRNA, and mRNA Green: down-regulation; Red: up-regulation; Gray: non-differential expression. Upsidedown triangle: circRNA, diamond: miRNA, rectangle: gene, triangle: lncRNA.

Based on the information from miRanda and TargetScan, 309 miRNAs were predicted to interact with 206 DE cicRNAs, among which 5 miRNAs were differentially expressed. Visual representation of circRNA-miRNA-mRNA interactions was conducted for 5 DE miRNAs, while DE lncRNAs that play regulatory roles in DE-genes were integrated. As shown in Figure 3, an interaction network comprising 362 edges connecting 339 nodes was established and contained 5 miRNAs (3 up-miRNA and 2 down-miRNA), 41 circRNAs (20 up-circRNA and 21 down-circRNA, 82 lncRNAs (82 down-lncRNs), and 211 genes (47 down-genes and 164 non-DE genes). Five miRNAs, miR-582-5p, miR-665, miR-1197, miR-657, and miR-582-3p, were hub nodes with highly connected degrees (Table 1).

**Table 1:** Top 10 nodes with high connective degree in ceRNA network

ID	DE	degree
hsa-miR-582-5p	UPMIR	123
hsa-miR-665	DOWNMIR	55
hsa-miR-1197	DOWNMIR	36
hsa-miR-657	UPMIR	31
hsa-miR-582-3p	UPMIR	31
ADAMTSL1	DOWNGENE	17
CTNND2	DOWNGENE	12
DCX	DOWNGENE	8
FOXP2	DOWNGENE	8
ROBO2	DOWNGENE	7

DE: differentially expression

### Pathway enrichment analysis for significant miRNAs

**Figure 4:**
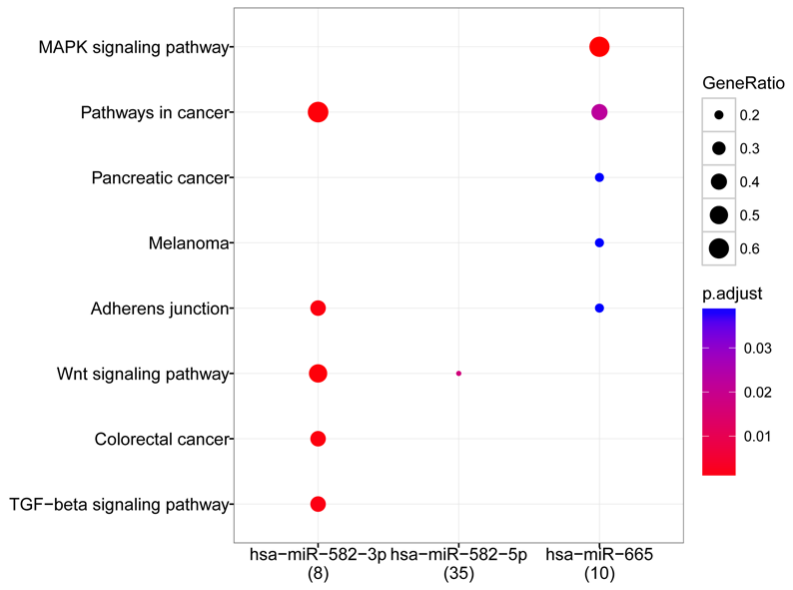
Bubble diagram of pathways related to hub miRNAs

The 5 hub miRNAs were subjected to pathway enrichment analysis, which revealed enriched pathways for 3 hub miRNAs: hsa-miR-582-3p, hsa-miR-582-5p, and hsa-miR-665. Hsa-miR-582-3p and hsa-miR-665 were closely related to pathways in cancer. Target genes of hsa-miR-582-3p and hsa-miR-582-5p were greatly enriched in the Wnt signaling pathway. Other significant pathways were found to be MAPK signaling pathways related to hsa-miR-665, adherens junction, and TGF-beta signaling pathway related to hsa-miR-582-3p (Figure 4).

### TF regulatory network

**Figure 5:**
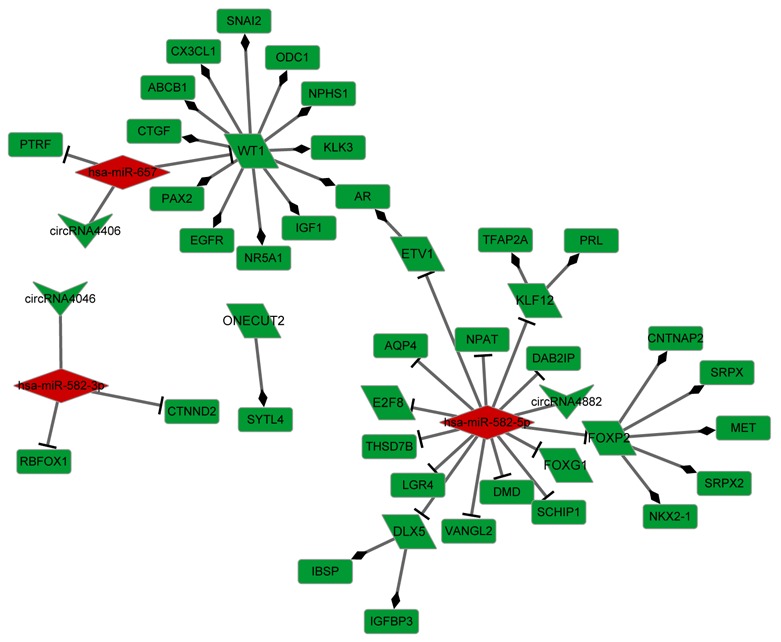
Transcriptional factor regulatory network Green: down-regulation, Red: up-regulation; Upside-down triangle: circRNA, diamond: miRNA, rectangle: gene, Oblique quadrilateral: transcriptional factor.

To further understand the role of the 3 significant miRNAs described above, miRNA target DE genes were subjected to the prediction of TF regulatory relationships revealed by the TRRUST database. We obtained 8 TFs (including *DLX5* (distal-less homeobox 5), *E2F8* (E2F transcription factor 8), *ETV1* (ETS variant 1), *FOXG1* (forkhead box G1), *FOXP2* (forkhead box P2), *KLF12* (Kruppel-like factor 12), *ONECUT2* (one cut homeobox 2), and *WT1* (Wilms tumor 1) among the 47 down-regulated genes in the ceRNA network (Figure 5). The TF regulatory network was constructed with 21 miRNA-target pairs and 23 TF-regulatory relationships. Three circRNAs (circRNA4046, circRNA4882, and circRNA4406) were included in the TF regulatory network as miRNA targets. Hsa-miR-657 target WT1 and hsa-miR-582-5p target ETV1 simultaneously regulated the *AR* gene.

### qRT-PCR validation

qRT-PCR analysis of hsa-miR-1197 and miR-665 expression was validated in the blood samples of patients and healthy controls. The expression level of hsa-miR-1197 was lower in patients with lung cancer than in normal controls. The expression of hsa-miR-665 was up-regulated in the patient group compared to that in the controls. The expression profile of hsa-miR-1197 and miR-665 was similar to the RNA-seq findings.

## DISCUSSION

Cross-talk between ceRNAs based on shared miRNAs plays key roles in the physiology and development of various cancers [11]. Lung cancer is the most common cancer type and the leading cause of cancer-related death worldwide. Genetic factors are the strongest contributors to cancer development. However, transcriptome changes in lung cancers remain unclear. RNA-seq is a newly developed transcriptome profiling technology that is widely applied to detect new genetic variants and quantify genomic expression in a specific assay.

In the previous study, Jiao Yuan et al performed microarray data analysis covering both mRNAs and lncRNAs of four tumor types of gastric, colon, liver and lung cancer and found 316 DE genes and 157 DE lncRNAs [12]. To understand the role of non-coding RNA in lung cancer development, we performed RNA-seq (miRNA- and circRNA-seq) using blood samples of patients with lung cancer and controls. The expression profiles of mRNAs, miRNAs, circRNAs and lncRNAs were obtained for lung cancer samples. Our data showed that a total of 59 miRNAs, 292 circRNAs, 232 lncRNAs, and 18,306 mRNAs were differentially expressed in lung cancer samples. Based on miRNA profiling, miR-1253, miR-504, and miR-26a-5p were found to be the biomarkers for predicting prognosis in non-small cell lung cancer [13]. Another RNA-seq analysis showed that miR-218 targeting N-cadherin was down-regulated in aggressive lung cancer adenocarcinoma cells [14]. All these above indicated the significant role of miRNAs in the lung cancer development.

Similarly, the ceRNA network established in this study also revealed 5 hub miRNAs, namely hsa-miR-582-5p, hsa-miR-665, hsa-miR-1197, hsa-miR-657, and hsa-miR-582-3p. Among the 47 miRNA target DE genes, 24 were documented to be related to lung cancer, which contributed to the significance of miRNAs in the ceRNA network.

KEGG pathway analysis revealed functional enrichment of hsa-miR-582-3p, hsa-miR-582-5p, and hsa-miR-665 in pathways in cancer, including pancreatic cancer, melanoma, and colorectal cancer, which validated the significant status of these miRNAs in cancer development. Additionally, hsa-miR-582-3p and hsa-miR-582-5p were significant nodes in the TF regulatory network and relevant to the Wnt signaling pathway. The critical role of the Wnt signaling pathway in lung carcinogenesis has been demonstrated previously [15, 16]. Up-regulation of Wnt pathway components is related to late stage and poor prognosis of non-small cell lung cancer [16]. The alteration of miR-582-3p expression was shown to be correlated with poor prognosis of patients with non-small cell lung cancer by activating Wnt/β-catenin signaling [17]. Furthermore, a new evidence has shown that the Wnt-β-catenin signaling pathway has function in regulating the expression of telomerase subunit *Tert* *[18]**.* Telomerase is a most active enzyme in tumors, which controls telomere length and contributes to tumorigenesis [19]. Telomerase has been proposed to be the target for cancer therapy [20]. The dysregulation of β-catenin could lead to the overexpression of *Tert,* stabilize the telomeres, and further results in tumor formation. Intervention of Wnt/β-catenin signaling regulated by hsa-miR-582-3p and hsa-miR-582-5p may be associated with the telomerase activity, which can be used to discover the new antitelomerase therapy for lung cancer. Hsa-miR-582-5p has recently been investigated in various cancers, such as bladder cancer [21] and liver cancer [22]. Hsa-miR-582-5p was found to be significantly down-regulated in high-grade bladder cancer, while the accumulation of miR-582-5p suppressed tumor growth [21]. A similar expression pattern of miR-582-5p was observed in liver tumors and contributes to cancer progression by targeting the cyclin-dependent kinase 1 and AKT serine/threonine kinase 3 genes [22]. However, in this study, miR-582-5p was greatly up-regulated in the blood of patients with lung cancer. ETV1 is a DE TF regulated by miR-582-5p, which was recently proposed as an oncogene in non-small cell lung cancer [23]. Furthermore, WT1 as a Wilms’ tumor gene was found to be overexpressed in various types of cancers [24]. We found that WT1 as a DE TF was down-regulated by hsa-miR-657. Whether the different findings can be attributed to sample variance requires further analysis.

MiRNAs are discovered as a class of small noncoding RNAs that inversely regulate gene expression [25]. Our results showed that target genes of up-regulated miRNAs were greatly associated with cancer-related pathways such as pathways in cancer, including colorectal cancer, and choline metabolism in cancer. Similar findings were observed in the DE gene pathway analysis; central carbon metabolism in cancer was altered by up-regulated genes. These results indicate the reliability of our findings. Additionally, two novel pathways of olfactory transduction and neuroactive ligand-receptor interactions were dysregulated by DE genes.

Olfactory transduction is induced by high concentrations of odorants mediated by adenylate cyclase [26]. Cyclin AMP (cAMP) is produced by adenylate cyclase, which is a second messenger in olfactory transduction. The expression of cAMP is elevated in patients with lung cancer [27]. Our pathway enrichment analysis also indicated that the cAMP signaling pathway was associated with DE miRNAs, suggesting that the cAMP signaling pathway may be dys-regulated in cancer progression. Previous studies showed that cAMP response element-binding protein (CREB) regulated the expression of genes involved in cell apoptosis, proliferation, and inflammation [28]. CREB contributes to cancer cell growth and metastasis. Overexpression of CREB was found in lung cancer cases with poor prognosis [29]. Thus, CREB may be targeted for the development of lung cancer therapies. In the present study, olfactory transduction was significantly enriched by the olfactory receptor (OR) gene, such as *OR13C3*, *OR56A4*, and *OR1J2*. Somatic mutations in *OR13C3* and *OR1J2* were detected in pancreatic tumors. Although the direct evidence for the correlation between the olfactory transduction pathway and lung cancer is rare, outside signals may be transmitted to cells through olfactory transduction underlying tumorigenesis.

Genes significantly contributing to the neuroactive ligand-receptor interaction pathway are highly relevant to lung cancer and have diverse functions, such as *CHRNA3* (cholinergic receptor nicotinic alpha 3 subunit), *CHRNA5*, *GRPR* (gastrin-releasing peptide receptor), *SSTR2* (somatostatin receptor 2), and *CHRNB4* (cholinergic receptor nicotinic beta 4 subunit). *CHRNA3*, *CHRNA5*, and *CHRNB4* are subunits of the nicotinic acetylcholine receptor, which contribute to lung cancer risk [30]. A previous study showed that single-nucleotide polymorphisms in *CHRNA5* and *CHRNA3* were associated with nicotine dependence [31]. *GRPR* has been found to be highly expressed in patients with lung cancer induced by nicotine, which stimulates cell proliferation, contributing to tumorigenesis [32]. Additionally, *SSTR2* has been proposed as a therapeutic candidate for neuroendocrine tumors, such as small cell lung cancer [33]. Thus, the neuroactive ligand-receptor interaction pathway may be stimulated by the response to nicotine and involved in neuroendocrine processes.

In this paper, we used the pooled samples for analysis, which may ignore the differences between individuals. However, in this study we attempted to analyzed the common molecular mechanism throughout all types of lung cancers. Thus, studies with a large sample size are warranted.

Crosstalk between miRNAs, mRNAs, circRNA, and lncRNAs reveals the complex mechanism underlying lung cancer development and progression. Significant miRNAs play pathological roles in tumorigenesis of lung cancer, such as miR-582-5p, miRNA-582-3p, and miR-657. The aberrant regulation of key miRNAs in ceRNA network may also perturb the pathways of olfactory transduction and neuroactive ligand-receptor interactions in lung cancer development. Our work may provide the novel perspective to understanding the pathogenesis underlying lung cancer and pave the way to uncover new target therapy for lung cancer.

## MATERIALS AND METHODS

### Patients and sampling

Approval was obtained from the ethics committee of the First Hospital of Jilin University and all participants provided informed consent before the study. A total of 38 patients with lung cancer and 23 age-paired normal individuals were enrolled in the study between January 2016 and January 2017. All patients were histopathologically diagnosed with adenocarcinoma, squamous carcinoma, or other types of lung cancer. The healthy controls who underwent physical examinations in our hospital were included in this study. The basic characteristics of the enrolled subjects were listed in Supplementary Table 1. Total 18 paired peripheral blood samples (3 mL) from patients and healthy controls were collected after 12 h fasting at the First Hospital of Jilin University. Samples from 3 patients with similar pathological characteristics were pooled together. Peripheral blood mononuclear cells were isolated from 12 pooled blood samples in the patient group (n = 6) and controls (n = 6) by Ficoll-Hypaque density separation (Flow Laboratories, Irvine, UK). Total RNA was isolated from fresh collected peripheral blood mononuclear cells using an RNeasy mini kit according the manufacturer’s instructions (Qiagen, Hilden, Germany). The purity and integrity of the RNA samples were measured by a Nanodrop spectrophotometer (Wilmington, DE, USA) and Agilent 2100 Bioanalyzer (Santa Clara, CA, USA).

### miRNA sequencing (miRNA-seq)

Six pooled samples from the patient and control groups were used for miRNA-seq. The detailed information for the pooled samples is shown in Supplementary Table 1. A small RNA-seq library was constructed by using TruSeq™ Small RNA Sample Prep Kits (Illumina, San Diego, CA, USA) according to the manufacturer’s instructions. Total RNA (1 μg) was ligated with 3′ and 5′ Adenylated Adapter, followed by reverse transcription reactions. Complementary DNA (cDNA) was amplified by PCR and the products were purified on a 6% polyacrylamide Tris-borate-EDTA gel. Next, the library preparations were subjected to next-generation sequencing on an Illumina Hiseq2000/2500 platform with 150-base pair single-end reads.

The quality of raw reads was controlled by the FastQC tool [34]. Unreliable bases and low-quality reads were removed by prinkitseq-lite [35] and fastx toolkit [36]. The clean data were mapped to the reference human miRNA gene database provided by GENCODE [37] using StringTie software [38].

### CircRNA sequencing (circRNA-seq)

Another 3 RNA specimens from each group were subjected to RNA-seq (Supplementary Table 1). An Epicentre Ribo-Zero*™* kit (Illumina) was used to remove rRNA from total RNA (3 μg) according to the manufacturer’s instructions. After purification, the depleted RNA was fragmented and used for first-strand cDNA synthesis with random hexamer primers followed by second-strand synthesis with a dUTP mixture. The purified double-stand DNA products were treated with T4 DNA polymerase and Klenow DNA polymerase, and then ligated with adaptors. The circRNA library was constructed by PCR amplification with first-strand cDNA selected by AMPureXP beads (Beckman Coulter, Brea, CA, USA) and sequenced on the Illumina Hiseq4000 platform with 150-base pair paired-end read generation.

Valid reads were obtained after the raw dataset was preprocessed by the FastQC and RseQC quality control tool. Genomic circRNA were mapped to the human reference genome (GRCh38) by TopHat2 [39]. Next, all circRNAs were annotated for circRNA-hosting genes with the application GENCODE v24 [40]. Protein-coding genes and lncRNAs were collected for further analysis.

### Differential analysis of patients and controls

DE miRNAs between patients and controls were detected by the limma package in R. P values were controlled by Benjamini and Hochberg’s false discovery rate procedure. miRNAs with |fold-change (FC)| > 2 and false discovery rate < 0.05 were considered differentially expressed in lung cancer cases.

Based on the circRNA-seq dataset, the genes (mRNAs) and lncRNAs showing differential expression (|log2FC (fold-change)| > 1 and q value < 0.05) were analyzed by Cuffdiff of the Cufflinks package [41]. DEseq package in R was used to detect circRNAs at differential expression levels (p value < 0.05).

### miRNA–target prediction and function annotation

miRNA-target gene interactions were predicted based on seven publicly available databases. Conserved miRNA targets recoded in miRecords [42] and MiRWalk database [43] were collected and those deposited in at least three databases of miRanda [44], MirTarget2 [45], PicTar [46], PITA [47], TargetScan [48] were filtered. The two parts of predicted targets were integrated by screening overlapping miRNA targets. Subsequently, the miRNA targets were subjected to Kyoto Encyclopedia of Genes and Genomes (KEGG) pathway analysis by using clusterProfiler R package [49]. Similarly, DE genes were subjected to pathway analysis at a significance level of P < 0.05.

### CeRNA network analysis

TargetScan 7.0 Human database (http://www.targetscan.org/) [50] is a collection of conserved target sites of conserved miRNA families. miRanda [44] predicts miRNA targets by scoring the binding energy of the miRNA to the targets. Potential interactions between circRNA and miRNA were predicted by TargetScan 7.0 and miRanda based on miRNA-target gene interactions. In miRanda analysis, the threshold miRanda-type score was set as ≤ -20 with other default parameters. Based on the miRNA-target gene interactions obtained above, a circRNAs-miRNAs-mRNA network was constructed and visualized with Cytoscape software [51]. LncRNAs that play regulatory roles in DE genes were filtered and integrated into the ceRNA network.

### Pathway analysis of significant miRNAs

Based on the topology of the ceRNA network, hubs with a high degree centrality were screened. miRNAs showing a high degree in the ceRNA network were subjected to pathway enrichment analysis. ClusterProfiler was utilized to classify pathway terms for miRNA target gene clusters. A pathway was considered significant when P < 0.05.

### TF regulatory network analysis

The Transcriptional Regulatory Relationships Unraveled by Sentence-based Text mining (TRRUST) is a collection of literature-curated TF-target interactions. The transcriptional regulation interaction between DE TFs and targets were retrieved from the TRRUST database. Based on the miRNA-target interactions (including miRNA-circRNA and miRNA-gene interactions), the TF regulatory network was constructed by connecting miRNAs, TFs, circRNAs, and DE genes.

### Quantitative reverse transcription-polymerase chain reaction (qRT-PCR) detection of significant miRNAs and target genes

We verified the differential expression of hsa-miR-1197 and hsa-miR-665 in the patient group compared to that in healthy controls. qRT-PCR analysis was conducted using blood RNA samples from the remaining 20 patients with lung cancer and 5 healthy controls. The primers used in this analysis were synthesized by Sangon Biotech (Shanghai, China) and the primer sequences were as follows: hsa-miR-1197 forward: GCAGGACACATGGTCTACTTCT, reverse: GCTGTCAACGATACGCTACCTA; hsa-miR-665 forward: ACCAGGAGGCTGAGGCCCCTAA, reverse: GCTGTCAACGATACGCTACCTA; GAPDH forward: TGACAACTTTGGTATCGTGGAAGG and reverse: AGGCAGGGATGATGTTCTGGAGAG. The reverse transcription reaction was performed using PrimeScript™ RT Master Mix (RR036A, TAKARA, Shiga, Japan) for cirRNAs and miRNA First-Strand cDNA Synthesis Kit (EY001, Eryun, Shanghai, China) for miRNAs. PCR amplification was achieved under Applied Biosystems® ViiA™ 7 PCR system (Foster City, CA, USA). The expression value of miRNA, circRNA, and genes was calculated by the 2^−ΔΔCT^ method relative to GAPDH.

### Statistical analysis

Statistical analysis was performed using SPSS 22.0 (SPSS, Inc., Chicago, IL, USA). Data were expressed as the mean ± SEM (standard error of the mean). Difference between groups were compared by *t* test or Mann-Whitney. P < 0.05 was considered statistically significant.

## SUPPLEMENTARY MATERIALS TABLE





## Abbreviations

(miRNAs): MicroRNAs
(mRNAs): messenger RNAs
(ceRNA): competing endogenous RNA
(TFs): transcription factors
(circRNAs): Circular RNAs
(RNA-seq): RNA sequencing
(DE): Differentially expressed
(*E2F8*): E2F transcription factor 8
(*ETV1*): ETS variant 1
(*FOXG1*): forkhead box G1
(*FOXP2*): forkhead box P2
(*KLF12*): Kruppel-like factor 12
(*ONECUT2*): one cut homeobox 2
(*WT1*): Wilms tumor 1
(cAMP): Cyclin AMP
(CREB): cAMP response element-binding protein
(OR): olfactory receptor
(*CHRNA3*): cholinergic receptor nicotinic alpha 3 subunit
(*GRPR*): gastrin-releasing peptide receptor
(*SSTR2*): somatostatin receptor 2
(*CHRNB4*): cholinergic receptor nicotinic beta 4 subunit
(KEGG): Kyoto Encyclopedia of Genes and Genomes
(TRRUST): Transcriptional Regulatory Relationships Unraveled by Sentence-based Text mining
(qRT-PCR): Quantitative reverse transcription-polymerase chain reaction
