# Supplementary material for: Crosstalk in competing endogenous RNA network reveals the complex molecular mechanism underlying lung cancer
Source: Oncotarget. 2017 Aug 24;8(53):91270–80. doi: 10.18632/oncotarget.20441 (PMC5710922; doi:10.18632/oncotarget.20441)
Supplement: Supplementary file 1 [file oncotarget-08-91270-s001.pdf]

Crosstalk in competing endogenous RNA network reveals the complex molecular mechanism underlying lung cancer

SUPPLEMENTARY MATERIALS

Supplementary Table 1: The basic information of subjects included in RNA-seq analyses.

See Supplementary File 1
